# Naming Matters: Hydridic Hydrogen Atoms are Halogen, Chalcogen, and Pnictogen Bond Acceptors not “Hydridic Hydrogen Bond” Donors

**DOI:** 10.1002/chem.202502074

**Published:** 2025-08-13

**Authors:** Rosa M. Gomila, Roberta Beccaria, Cristina Lo Iacono, Antonio Frontera, Giuseppe Resnati

**Affiliations:** ^1^ Department of Chemistry Universitat de les Illes Balears Crta de Valldemossa km 7.5 Palma de Mallorca Baleares 07122 Spain; ^2^ NFMLab, Dept. Chemistry, Materials, Chemical Engineering “Giulio Natta” Politecnico di Milano Via L. Mancinelli 7 Milano I‐20131 Italy

**Keywords:** chalcogen bonds, halogen bonds, hydrogen bonds, pnictogen bonds, tetrel bonds

## Abstract

The definition and classification of noncovalent interactions are essential for consistent communication in supramolecular chemistry. A recent paper introduced the term “hydridic hydrogen bond” to describe interactions where hydrogen atoms bound to electropositive elements (e.g., Si, Ge, Sn) act as electron donors. However, such terminology conflicts with IUPAC definition, which requires hydrogen to be bonded to more electronegative atoms for a hydrogen bond (HB). Herein, we combine electrostatic potential analysis, energy decomposition, ELF/QTAIM, and NBO calculations to show that interactions involving hydridic hydrogen atoms as donors and heavier main group elements (Br, Se, As) as acceptors are better classified as halogen, chalcogen, and pnictogen bonds (PnBs), respectively. Our results confirm the nucleophilic character of hydridic hydrogen atoms and the electrophilic role of the interacting elements via σ‐ and π‐hole interactions, consistent with established IUPAC definitions. Experimental evidence from NMR supports this classification. By clarifying these interactions’ nature and directionality, we advocate for nomenclature that aligns with chemical intuition and community standards, thereby avoiding confusion caused by unnecessary redefinitions.

## Graphical abstract

Hydridic hydrogen atoms (e.g., in Me_3_Ge─H) form directional noncovalent interactions with electrophilic Br, Se, and As centers. Through electrostatic and orbital analyses, these interactions are identified as halogen, chalcogen, and PnBs, not as “hydridic hydrogen bonds.” The study promotes clarity in nomenclature consistent with IUPAC guidelines.

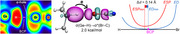

## 1 Introduction

Noncovalent interactions are of paramount importance throughout chemistry, biology, and physics, as they determine aggregation, recognition, and self‐assembly of molecules. The hydrogen bond (HB) is by far the most impactful interaction. Acknowledging a perspective that predates even the seminal work of Latimer and Rodebush,^[^ ^1^ ^]^ the recent IUPAC definition states that an HB involves “a hydrogen atom from a molecule or a molecular fragment X─H, in which X is more electronegative than H.”^[^ ^2^ ^]^ These hydrogen atoms have a partial positive charge, act as electrophiles (Lewis acids), and attractively interact with nucleophiles (Lewis bases). There are many other interactions wherein partially positive atoms function as electrophiles/Lewis acids and attractively interact with nucleophiles/Lewis bases.

Adopting a nomenclature consistent with that of the HB, where the name refers to the electrophilic component, various electrophile/nucleophile interactions are similarly named after the electrophilic element involved. For example, tetrel (TtB),^[^ ^3^ ^]^ triel (TrB),^[^ ^4^ ^]^ coinage (CiB),^[^ ^5^ ^]^ or matere (MaB)^[^ ^6^ ^]^ bonds are the weak bonds where the electrophile is an element of group 14, 13, 11, or 7. Following this practice, three IUPAC definitions establish that the halogen bond (HaB),^[^ ^7^ ^]^ chalcogen bond (ChB),^[^ ^8^ ^]^ and pnictogen bond (PnB)^[^ ^9^ ^]^ are the interactions wherein elements of groups 17, 16, and 15 function as electrophiles/Lewis acids and attractively interact with nucleophiles/Lewis bases. Having one or more lone pairs, the elements of the groups 17‐15 frequently function as nucleophiles, but when covalently bonded to electron‐withdrawing atoms/groups they also show an electrophilic character. The above cited IUPAC definitions explicitly mention iodoperfluoroalkanes and thiocyanates as prototype HaB and ChB donors and fluorine or chlorine atoms and cyano or oxygen groups as substituents that effectively promote the PnB formation.

In a recent study, Lamanec et al.^[^ ^10^ ^]^ reported that in molecules of the type X─H, the hydrogen atom bears a partial negative charge when X = Si, Ge, or Sn. This is consistent with Pauling electronegativity values, where H (2.20) is more electronegative than Si (1.90), Ge (2.01), and Sn (1.96).^[^ ^11^ ^]^ Upon forming X─H···Y─Z_n_ adducts (with Y = B, C, N, P, S, Br, I, and Z = CN, CF_3_, O, F, Cl), the X─H stretching frequencies show red shifts and intensity increases, spectroscopic features often associated with hydrogen bonding.

Interestingly, similar IR signatures are observed in traditional X─H···Y─Z_n_ adducts, where X is more electronegative than H (e.g., X = C, N, O, S, F, Cl, Br, I), and the hydrogen atom is partially positive. Lamanec et al.^[^ ^10^ ^]^ refer to these two categories as “protonic” and “hydridic” hydrogen bonds (HBs), respectively, noting that both display IR fingerprints typical of H‐bond formation. However, it is well recognized that such IR features are neither necessary nor sufficient for establishing the presence of a HB.^[^ ^12^ ^]^ The IUPAC definition requires that the hydrogen be covalently bonded to an atom more electronegative than itself, which excludes “hydridic” H atoms by definition.

Although Lamanec et al. acknowledge a nomenclature issue with applying the IUPAC HB definition to both cases, their proposed solutions involve rebranding rather than clarifying. In contrast, we argue that established IUPAC definitions and community‐accepted terminology already suffice to describe these interactions clearly and consistently, without invoking a new category of “hydridic HBs.”

Specifically, we show that interactions of the type X─H···Y─Z_n_, where X = Si, Ge, or Sn (i.e., elements less electronegative than hydrogen) and the H atom is nucleophilic, originate from σ‐ or π‐hole regions on the Y atom. Depending on the identity of Y, these interactions are more accurately classified using established terminology: as HaBs when Y is Br or I, ChBs when Y is S or Se, PnBs when Y is N or P, triel bonds (TrBs) when Y is B, and tetrel bonds (TtBs) when Y is C. Through detailed computational analyses and support from experimental observations, we argue that these classifications are not only chemically sound but also consistent with IUPAC definitions, thereby eliminating the need for an oxymoron, that is, “hydridic hydrogen bonds.” This approach preserves clarity and continuity in the nomenclature of noncovalent interactions, consistent with the recent proposal of a taxonomy of chemical interactions.^[^ ^13^ ^]^

## 2 Results and Discussion

We have selected some of the X─H···Y─Zn complexes studied by Lamanec et al.^[^ ^10^ ^]^ in which a hydridic H atom is involved, and we analyzed them using various computational tools (see computational methods in the Supporting Information). Me_3_GeH was chosen for X─H (electron density donor) and As(CN)_3_, Se(CN)_2_, and BrCN for Y─Z_n_ (electron density acceptors) for the σ‐hole complexes,^[^ ^14^ ^]^ and BF_3_, F_2_CO, and FNO_2_ for Y─Z_n_ (electron density acceptors) for the π‐hole complexes.^[^ ^15^ ^]^

**Figure 1:**
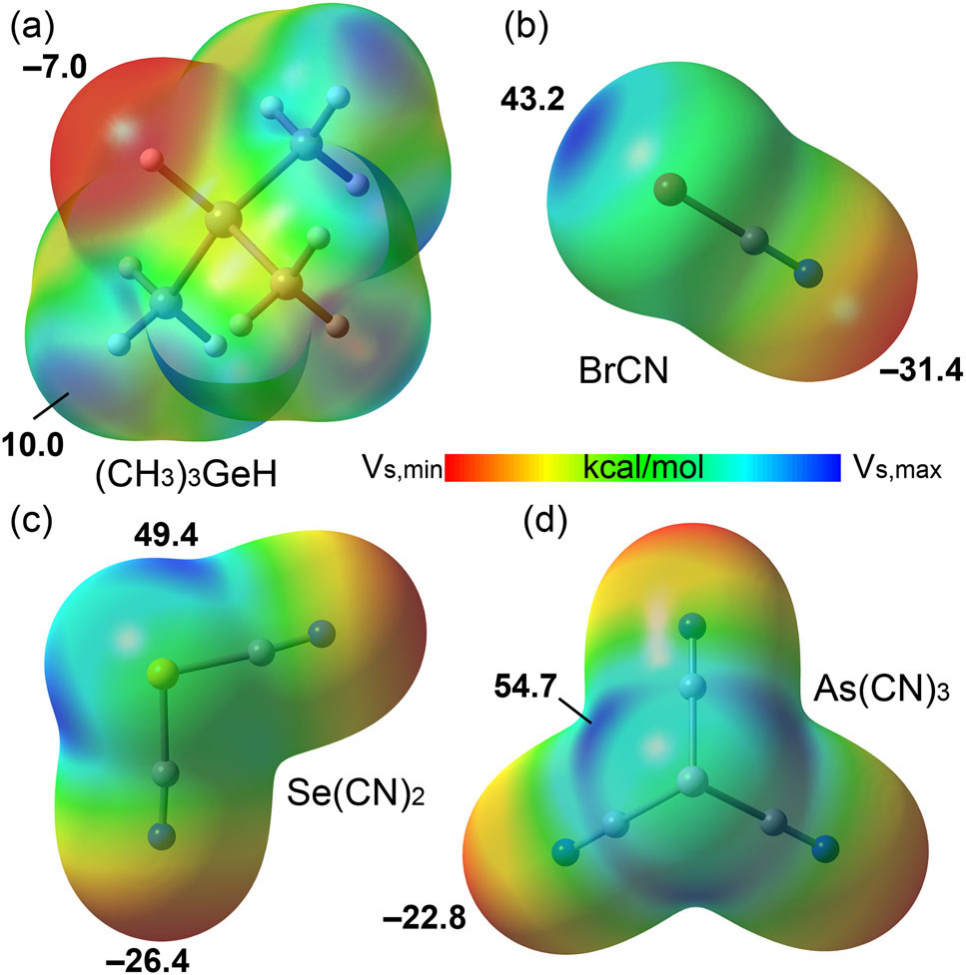
MEP surfaces of Me_3_GeH (a), BrCN (b), Se(CN)_2_ (c), and As(CN)_3_ (d). Energies in kcal/mol. The MEP minima and maxima are indicated (kcal/mol).

The MEP surface plots are shown in Figure 1 for the electron donor and acceptors forming σ‐hole complexes and in the Supporting Information (Figure S1) for the electron acceptors forming π‐hole complexes. Plots of Figure 1 clearly show that the H atom bonded to Ge is negative (V_S,max_ = −7.0 kcal/mol), that is, tailored to act as a nucleophile, whereas Br, Se, and As are significantly positive (MEP values ranging from 54.7 kcal/mol for As(CN)_3_ to 43.2 kcal/mol for BrCN)), that is, tailored to act as electrophiles. This straightforward analysis already indicates that, as required by IUPAC definitions, the formation of Ge─H···Y (Y = Br, Se, and As) complexes is driven by HaBs, ChBs, and PnBs, not HBs.

**Figure 2:**
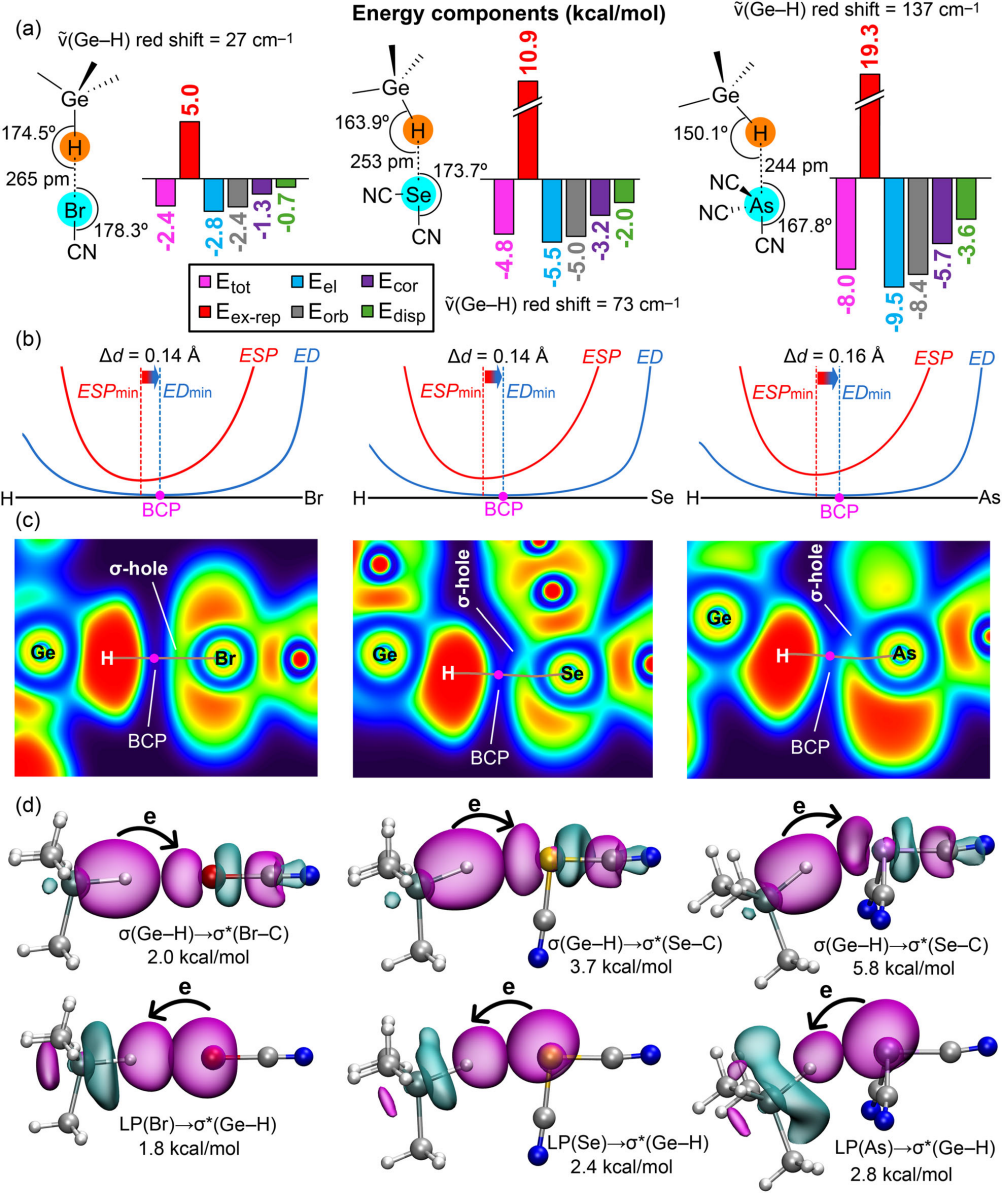
a) Chemical drawing of the complexes with the geometric features with indication of the Ge─H···Y and H···Y─CN distances and the Ge─H···Y and H···Y─CN angles in the optimized complexes. The IR shift of the Ge─H stretching wavenumbers is indicated. EDA analysis of the halogen (left), chalcogen (middle), and pnictogen (right) complexes with indication of the total (E_tot_, pink bar), exchange repulsion (E_ex‐rep_, red bar), electrostatic (E_el_, blue bar), orbital (E_orb_, grey bar), correlation (E_cor_, violet bar), and dispersion (E_disp_, green bar) terms. b) ED vs. ESP plots along the path connecting the H and Y (Y = Br, Se, and As) in the halogen (left), chalcogen (middle), and pnictogen (right) complexes with indication of the BCP in fuchsia. The x‐axis represents the bond path, while the y‐axis, omitted for clarity, denotes the values of electron density and electrostatic potential in atomic units. This axis has been truncated at 1.0 a.u. c) 2D ELF plots (blue and green regions have lower electron density; red and yellow regions have higher electron density); and QTAIM analysis (BCP in fuchsia and bond path in brown) of the halogen (left), chalcogen (middle), and pnictogen (right) complexes. d) NBO plots of the donor and acceptor orbitals characterizing the Ge─H···Y─CN contacts of the halogen (left), chalcogen (middle), and pnictogen (right) complexes. The E^(2)^ energies are indicated.

To confirm this assignment, we have optimized the σ‐hole complexes without imposing symmetry constraints and analyzed them using various computational methods, including energy decomposition analysis (EDA), electron localization function (ELF) combined with quantum theory of atoms in molecules (QTAIM), electron density (ED) versus electrostatic potential (ESP) along the bond path connecting the interacting atoms, and natural bond orbital (NBO) analysis (see Supporting Information for details). The results are summarized in Figure 2. The following key findings emerge from this analysis:

1. As dictated by the Br, Se, and As σ‐holes, the directionality of the interactions (Figure 2a) matches the characteristics of HaBs, ChBs, and PnBs. That is, the H···Y─CN angle (Y = Br, Se, As) is close to 180° as required in respective IUPAC definitions and is opposite to that typically observed in HBs, wherein H should approach the lone pair(s) of Y. Additionally, the total binding energies follow the same trend as the σ‐hole MEP values, further supporting our rationalization.
2. In all complexes, the ED vs. ESP minimum comparison along the bond path reveals that the ESP minimum is closer to the H atom, while the ED minimum is nearer to the heavier element (see Figure 2b). This indicates that the nucleophile is the hydridic H atom, while the electrophile is the heavier element, fully consistent with the IUPAC definitions of HaB, ChB, and PnB and contrasting with the IUPAC definition of HB.
3. The ELF 2D plots combined with QTAIM analysis (Figure 2c) reveal the presence of a bond critical point (BCP) and bond path connecting the H and Y atoms (Y = Br, Se, As), wherein the bond path passes through the σ‐hole (regions with lower ELF values, shown in green) while avoiding areas of higher electron localization on the heavier elements (lone pairs, shown in red). This behavior is consistent with the characteristics of HaB, ChB, and PnB interactions and contrasts with those of HB.
4. The EDA analysis (Figure 2a) indicates that electrostatic (E_el_) and orbital (E_orb_) interactions are the dominant attractive terms in these complexes, with electrostatic being the largest attractive contributor. This is consistent with the MEP analysis in Figure 1 and the strong electrophilicity of the heavier elements. Given the relevance of the E_orb_ term, further analysis of orbital effects was performed using the NBO method, focusing on second‐order perturbation analysis and orbital charge transfer. The results reveal that all complexes exhibit a dominant σ(C─H) → σ*(Y─C) electron donation, characteristic of σ‐hole interactions, fully consistent with the IUPAC definition of HaB, ChB, and PnB. Additionally, a notable back‐donation is observed: LP(Y) → σ*(C─H). This back‐donation, in conjunction with the dominant σ(C─H) → σ*(Y─C) electron donation, explains the red shift and intensity increase observed in the Ge─H stretching frequency upon complex formation. The red shifts are given in Figure 2a, and they follow the back‐donation trend, further supporting this explanation.

**Figure 3:**
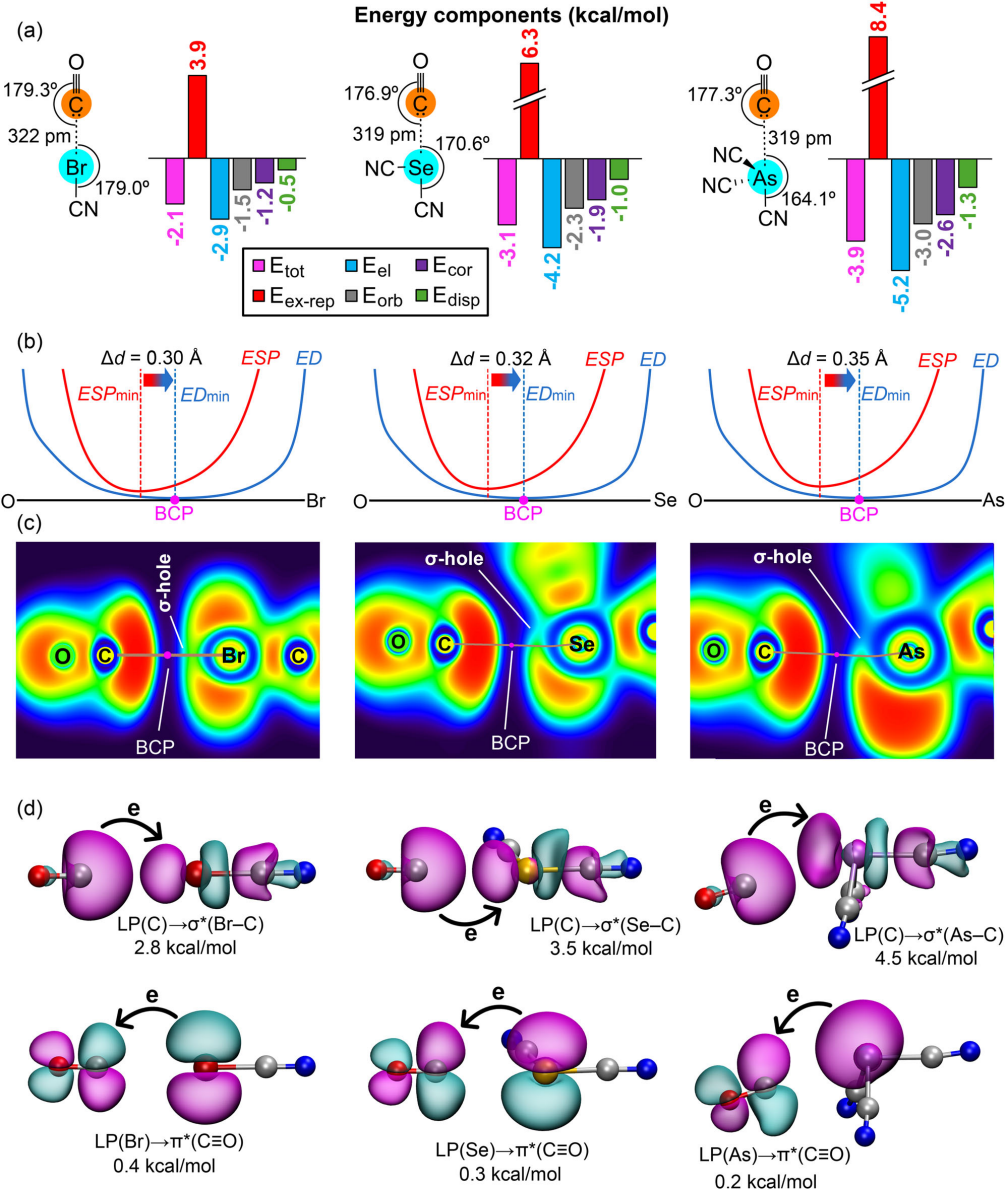
a) Chemical drawing of the CO complexes with the geometric features and indication of the OC···Y distances and the OC···Y─CN angles in the optimized complexes. EDA analysis of the halogen (left), chalcogen (middle), and pnictogen (right) complexes with indication of the total (E_tot_, pink bar), exchange repulsion (E_ex‐rep_, red bar), electrostatic (E_el_, blue bar), orbital (E_orb_, grey bar), correlation (E_cor_, violet bar), and dispersion (E_disp_, green bar) terms. b) ED vs. ESP plots along the path connecting the C and Y (Y = Br, Se, and As) in the halogen (left), chalcogen (middle), and pnictogen (right) complexes with indication of the BCP in fuchsia. The x‐axis represents the bond path, while the y‐axis, omitted for clarity, denotes the values of electron density and electrostatic potential in atomic units. This axis has been truncated at 1.0 a.u. c) 2D ELF plots (blue and green regions have lower electron density; red and yellow regions have higher electron density); and QTAIM analysis (BCP in fuchsia and bond path in brown) of the halogen (left), chalcogen (middle), and pnictogen (right) complexes. d) NBO plots of the donor and acceptor orbitals characterizing the OC···Y─CN contacts of the halogen (left), chalcogen (middle), and pnictogen (right) complexes. The E^(2)^ energies are indicated.

Analogous key finding can be extracted from the analysis of the π‐hole complexes included in the Supporting Information (Figures S1 and S2). The main difference with respect to σ‐hole ones is that the back‐donation was only observed in the pnictogen bonded π‐hole complex in the form π(N = O)→σ*(C─H). Germanium being less electronegative than silicon and tin, the conclusions drawn here for Me_3_GeH complexes can be reliably extended to analogous complexes formed by Me_3_SiH and Me_3_SnH.

To further strengthen our conclusions regarding the nature of hydridic interactions and provide clear comparisons with established σ‐hole bond systems, we have performed a parallel computational analysis using carbon monoxide (CO) as a typical lone pair donor molecule. This comparative study utilizes parallel analyses, including energy decomposition (EDA), minimum ED vs. ESP comparison along the bond path plots, topological descriptors (QTAIM and ELF), and NBO analysis. The aim is to provide a direct comparison between the characteristics of classical halogen, chalcogen, and pnictogen bonds formed with CO (Figure 3) and the hydridic interactions of Me_3_Ge─H complexes, the data for which are presented in Figure 2.

Remarkably, the results obtained with CO as a donor of electron density are highly analogous to those with (CH_3_)_3_GeH. For both sets of complexes, the linearity of the interactions (directionality) becomes progressively less pronounced from halogen to chalcogen to pnictogen (see Figure 3a), as typical for σ‐hole bonding.^[^ ^14^ ^]^ As expected, the OC···Y interaction distances are longer than the Ge─H···Y distances, attributable to the larger van der Waals radius of carbon compared to hydrogen. The partition energies derived from EDA not only exhibit comparable strengths but also show similar distributions of all energy terms. Electrostatic and orbital terms consistently dominate the attractive part, with exchange repulsion being the largest repulsive contribution. The ED versus ESP plots are also highly similar, albeit with larger Δd values, indicating analogous electronic features (see Figure 3b).

The ELF plots are likewise very similar (see Figure 3c), clearly showing a bond path connecting the interacting atoms by crossing the lone pair at carbon (a region of high electron density) and extending toward the σ‐hole in Y (a region of lower electron density), directly paralleling the behavior observed for (CH_3_)_3_Ge─H. Finally, the NBO analysis (see Figure 3d) yields very similar E^(2)^ values for the donation from the lone pair at carbon to the antibonding σ*(Y─C) orbital. The trend of these E^(2)^ values also parallels that observed for (CH_3_)_3_Ge─H, increasing from halogen to chalcogen and then to pnictogen bonding complexes. The main difference is the back‐donation that is negligible, ranging from 0.2 to 0.4 kcal/mol, due to the longer distances. Taken together, these consistent results further highlight how the features of interactions involving hydridic hydrogen atoms align with established σ‐hole bonding categories, reinforcing our proposed classification.

To further differentiate the nature of the hydridic interactions from conventional hydrogen bonds, we have performed a comparative computational analysis using hydrogen fluoride (HF) as a typical HB donor molecule. Our analyses reveal that, in sharp contrast to the σ‐hole‐driven interactions of Me_3_Ge─H with halogen, chalcogen, or pnictogen atoms (Figure 2), HF preferentially forms HBs with the nitrogen atom of the cyano substituent in the acceptor molecules (see Figure S3, Supporting Information). This selectivity is strongly supported by the MEP surface analysis (Figure 1), which clearly indicates the most negative electrostatic potential, and thus the most nucleophilic site for conventional HB acceptance, resides at these cyano nitrogen atoms. This distinct interaction pattern provides additional support that the hydridic hydrogen does not behave as a conventional HB donor seeking lone pair alignment but rather participates in interactions with the σ‐hole of the heavy element, aligning with the proposed classification as halogen, chalcogen, and PnBs.

Also, experimental findings support our standpoint. For iodoperfluoroalkanes, the shift to lower frequencies of ^19^F NMR signals of F atoms geminal to iodine is a fingerprint of HaB formation.^[^ ^16^ ^]^ Et_3_SiH and i‐Pr_3_SiH shift the signals of unfunctionalized perfluorocarbons to higher frequencies (Supporting Information), showing this is the “generic solvent effect” of these compounds (Table S1). Differently, signals of ─CF_2_I groups of a variety of mono‐ and diiodoperfluoroalkanes are shifted to lower frequencies (Tables S2, S3), and the shift values increase with the silane concentration, consistent with the presence of a HaB (Table S4). The observed upfield shifts are small; they are smaller than those seen when pyridine and amine derivatives interact with haloperfluoroalkanes.^[^ ^17^ ^]^ This is consistent with the typical trends observed in HaB formation, as the stronger the formed interactions are, the greater the resulting shifts are, and trialkylsilanes form, with iodo‐ and bromoperfluoroalkanes, interactions weaker^[^ ^18^ ^]^ than pyridine and amine derivatives.^[^ ^19^ ^]^

Our study presents a comprehensive analysis of noncovalent interactions involving hydridic hydrogen atoms as acceptors in halogen, chalcogen, and PnBs via σ‐holes, as well as TrB, TtB, and PnBs involving π‐holes. These findings challenge Lamanec et al.^[^ ^10^ ^]^ interpretation of these interactions as “hydridic hydrogen bonds” and demonstrate their alignment with IUPAC definitions, highlighting the electrophilic role of the heavier elements and the nucleophilic nature of hydridic hydrogen atoms. Furthermore, the investigation of the directionality, energetic contributions, and charge transfer mechanisms of the interactions as well as the experimental results reinforces this alignment with IUPAC definitions. These insights not only clarify the nomenclature of interactions but also prevent mistakes in the understanding of their nature.

## 3 Conclusions

In conclusion, the terminology used in scientific discussion carries profound implications for both clarity and progress in the field. The misnaming presented in Lamanec's work^[^ ^10^ ^]^ is not a minor inconsistency but a significant deviation from well‐established definitions, leading to potential confusion and misdirection in subsequent studies. For the discussed adducts, adherence to electronegativity, as required by the IUPAC definition of HB,^[^ ^2^, ^12^ ^]^ is essential and leads to unambiguous classifications. In contrast, relying on auxiliary features such as IR red shifts and intensity increases leads to inconsistent and potentially misleading nomenclature. Our computational analyses of IR shifts for halogen, chalcogen, and PnBs consistently show red shifts that follow the back‐donation trend predicted by NBO, further supporting their intrinsic spectroscopic signatures. Moreover, our parallel computational analysis using CO as a classical lone pair donor reveals highly analogous geometric and electronic features to the Me_3_Ge─H complexes, reinforcing their robust alignment with established σ‐hole bonding categories. Crucially, comparisons with a conventional hydrogen‐bond donor like HF demonstrate a distinct preference for cyano nitrogen as an acceptor and the persistent interaction of Me3Ge─H with the σ‐hole, providing a clear basis for rejecting hydrogen bonding as a descriptor. This aligns with broader logical^[^ ^13^ ^]^ and philosophical perspectives. As Bertrand Russell noted, “The first requisite of an ideal language would be that there should be one name for every simple, and never the same name for two different simples.”^[^ ^20^ ^]^ Similarly, Confucius emphasized the importance of precise language, stating, “If names be not correct, language is not in accordance with the truth of things.”^[^ ^21^ ^]^ These insights reinforce the necessity of clear and consistent terminology in scientific discourse.

## Supporting Information

Computational methods, Figures S1, S2 and S3 and Tables S1 to S4 with the ^19^F analyses are provided in the supporting information. The authors have cited additional references within the Supporting Information.^[^ ^22^, ^23^, ^24^, ^25^, ^26^, ^27^, ^28^, ^29^, ^30^, ^31^, ^32^, ^33^, ^34^ ^]^

## Conflict of Interest

The authors declare no conflict of interest.

## Supporting information



Supporting Information

## Data Availability

The data that support the findings of this study are available in the supplementary material of this article.
