# Supplementary material for: Naming Matters: Hydridic Hydrogen Atoms are Halogen, Chalcogen, and Pnictogen Bond Acceptors not “Hydridic Hydrogen Bond” Donors
Source: Chemistry. 2025 Aug 13;31(52):e02074. doi: 10.1002/chem.202502074 (PMC12444740; doi:10.1002/chem.202502074)
Supplement: Supplementary file 1 — Supporting Information [file CHEM-31-e02074-s001.docx]

**ESI for manuscript:**

**Naming Matters: Hydridic Hydrogen Atoms Are Halogen, Chalcogen, and Pnictogen Bond Acceptors Not “Hydridic Hydrogen Bond” Donors**

Rosa M. Gomila,^[a]^ Roberta Beccaria,^[b]^ Cristina Lo Iacono,^[b]^ Antonio Frontera,*^[a]^ Giuseppe Renati*^[b]^

**Table of contents:**

Page

1. Computational methods 2

2. Figures S1, S2 3

3. ^19^F NMR analyses 5

4. Cartesian coordinates and Thermochemical data at 298.15K and 0.1 MPa: 10

5. References 14

**1. Computational Methods.**

The full geometry optimizations without symmetry constraints and the energetic calculations were performed using the Turbomole 7.7 program^[22]^ at the PBE0-D4/aug-cc-pVTZ level of theory.^[23-25]^ The energy decomposition analysis (EDA) was conducted at the same level of theory, using the methodology implemented in Turbomole 7.7. Molecular electrostatic potential (MEP) surfaces were plotted using a 0.001 a.u. isosurface value. This level of theory has been validated in a benchmark study reported in the literature, providing results similar to CCSD(T)/def2-TZVP level of theory.^[26]^

The Electron Localization Function (ELF)^[27]^ and Quantum Theory of Atoms in Molecules (QTAIM)^[28]^ analyses were also performed at the same computational level. The Multiwfn program^[29]^ was employed to calculate ELF, QTAIM, and electron density (ED) versus electrostatic potential (ESP) analyses.^[30]^ This method, based on electronic criteria, allows for the unambiguous determination of electrostatically driven noncovalent bond types. According to the electronic criterion, the minimum of the ESP along the bond path is located closer to the atom donating electrons, while the minimum of the ED is closer to the atom presenting its electrophilic site for noncovalent bonding.

Natural Bond Orbital (NBO)^[31]^ analysis was performed using the NBO7 program,^[32]^ and the results were visualized with the VMD software.^[33]^

**2. Figures S1, S2**


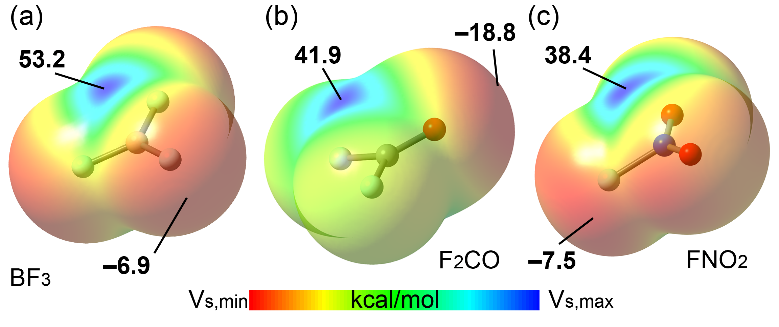


**Figure S1.** MEP surfaces of BF_3_ (a), F_2_CO (b) and FNO_2_ (d). Energies in kcal/mol. The MEP minima and maxima are indicated


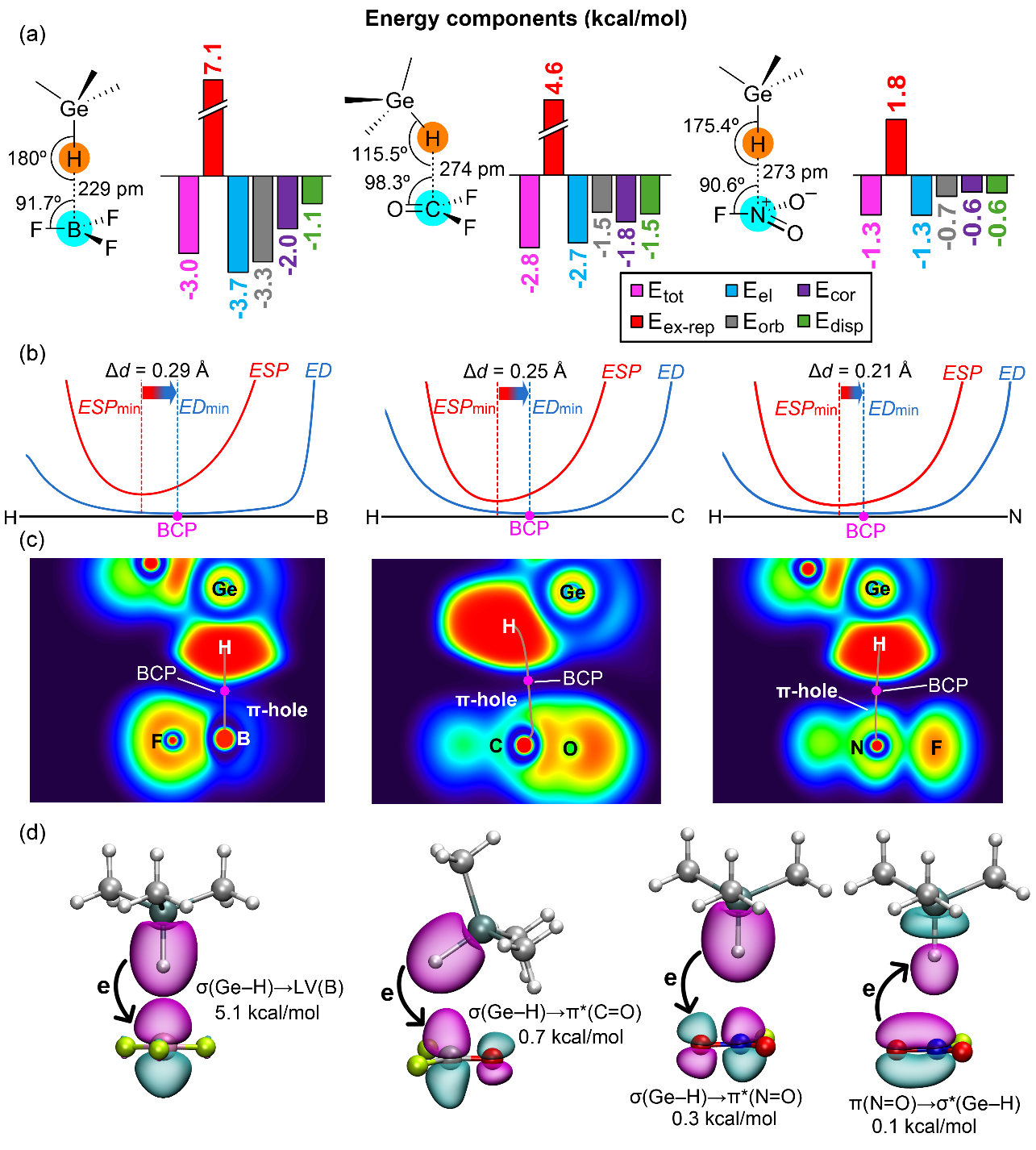


**Figure S2.** (a) Chemical drawing of the complexes with indication of the geometric features. EDA analysis of the triel (left), tetrel (middle) and pnictogen (right) complexes with indication of the total (E_tot_, pink bar), exchange repulsion (E_ex-rep_, red bar), electrostatic (E_el_, blue bar), orbital (E_orb_, grey bar), correlation (E_cor_, violet bar) and dispersion (E_disp_, green bar) terms. (b) ED vs ESP plots along the path connecting the H and Y (Y = B, C and N) in the triel (left), tetrel (middle) and pnictogen (right) complexes with indication of the bond critical point (BCP) in fuchsia. (c) 2D ELF plots and QTAIM analysis (BCP in fuchsia and bond path in brown) of the triel (left), tetrel (middle) and pnictogen (right) complexes; blue and green regions have lower electron density, red and yellow regions have higher electron density. The 2D planes are defined by the electron donor H-atom, the B,C,N electron acceptor and the F (for BF_3_ and NO_2_F) or O (for F_2_CO) atoms. (d) NBO plots of the donor and acceptor orbitals characterizing the Ge–H···Y contacts of triel (left), tetrel (middle) and pnictogen (right) complexes. The E^(2)^ energies are indicated.


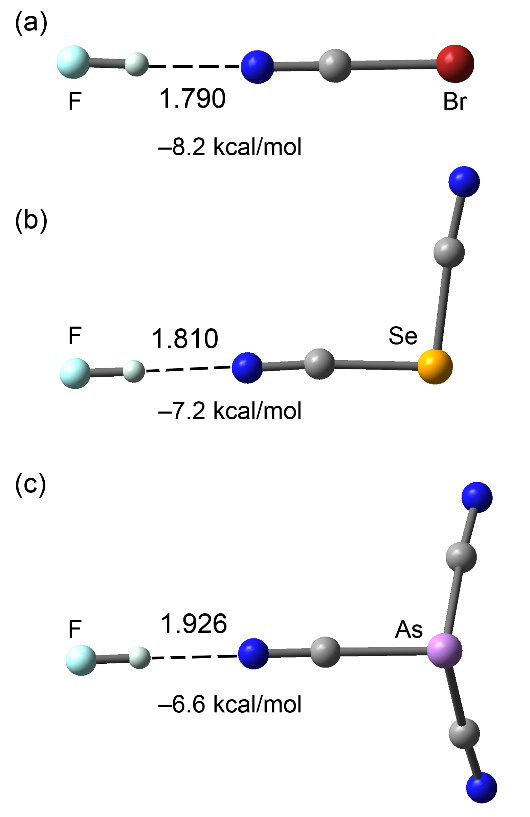


**Figure S3**. Optimized complexes of HF with BrCN (a), Se(CN)2 (b) and As(CN)3 (c). Distances in Å. Interaction energies are indicated.

**3. ^19^F NMR analyses**

Calculations indicate that hydrogen in trialkylsilanes are weaker donor of electron density and form weaker interactions with electrophiles than typical lone pair possessing atoms (e.g., nitrogen in pyridine). With the aim to maximize the formation of adducts with iodoperfluoroalkanes and the resulting NMR changes in ^19^F NMR studies, Et_3_SiH and *i*-Pr_3_SiH were used in large excess or as solvents. This is a benchmark method to identify the halogen bond formation.^[34]^ All four signals of *n*-perfluorohexane move to low fields in Et_3_SiH and *i*-Pr_3_SiH with respect to cyclohexane (used as reference and inert solvent)^[31]^ indicating that this is the shift caused by the unspecific solvent effect of trialkyl silane on perfluorocarbon moieties (Table S1). Similarly, all the signals of 1-iodoundecafluoropentane, 1-iodo-pentadecafluoroheptane, 1,8-diiodohexadecafluoroctane, 1,6-diiodododecafluorohexane, 1,4-diiodooctafluorobutane, and 1,2-diiodotetrafluoroethene show low-field shifts but the signal of the fluorine atoms geminal to iodine which show a high-field shift (Table S2). The same behaviour is observed if other saturated hydrocarbons (e.g, *n*-hexane, *n*-pentane, and cyclopentane) are used as reference inert solvents (Table S3) and the value of the shift decreases when the silane concentration decreases (Table S4). The formation of I···H–Si halogen bond is thus unquestionably proven.

**Methods.** The used compounds were purchased from ABCR or Merck and were used without further purification. ^19^F-NMR spectra were recorded at ambient temperature on Nuclear Magnetic Resonance Bruker NEO 500. All the chemical shifts are given in ppm with respect to C_6_F_6_ in d_6_-DMSO solution (internal capillary, -161.2900 ppm). Four decimal places given by the instrument pick-peaking are reported for the signals chemical shifts; chemical shift differences have been rounded to two decimal places.

**Table S1.** ^19^F NMR Chemical shifts of *n*-C_8_F_18_ in cyclohexane, Et_3_SiH, and *i*-Pr_3_SiH. The shifts of ^19^F signals in trialkylsilanes with respect to cyclohexane (Δδ=δ_cyclohexane_-δ_R3SiH_) are given in parenthesis.

| (**F–CF_2_CF_2_CF_2_CF_2_)_2_** | | | | |
| --- | --- | --- | --- | --- |
|  | **(*F*–C*F*_2_CF_2_CF_2_CF_2_)_2_** | **(F–CF_2_C*F*_2_CF_2_CF_2_)_2_** | **(F–CF_2_CF_2_C*F*_2_CF_2_)_2_** | **(F–CF_2_CF_2_CF_2_C*F*_2_)_2_** |
| ***c*-C_6_H_12_** | -82.3743 | -123.0767 | -123.8643 | -127.4204 |
| **Et_3_SiH** | -82.2405  (-.13) | -122.9557  (-.12) | -123.7476  (-.12) | -127.2921  (-.13) |
| ***i*-Pr_3_SiH** | -81.9629  (-.41) | -122.8923  (-.18) | -123.6661  (-.20) | -127.1616  (-.26) |

**Table S2.** ^19^F NMR Chemical shifts of I–(CF_2_)_5_F, I–(CF_2_)_7_F, I–(CF_2_)_2_–I, I–(CF_2_)_4_–I, I–(CF_2_)_6_–I, and I–(CF_2_)_8_–I in the three solvents of Table 1S. The shifts of ^19^F signals in trialkylsilanes with respect to cyclohexane (Δδ=δ_cyclohexane_-d_R3SiH_) are given in parenthesis.

| **I–(CF_2_)_5_F** | | | | | |
| --- | --- | --- | --- | --- | --- |
|  | **I–C*F*_2_** | **I–(CF_2_)C*F*_2_** | **I–(CF_2_)_2_C*F*_2_** | **I–(CF_2_)_3_C*F*_2_** | **I–(CF_2_)_4_C*F*_3_** |
| ***c*-C_6_H_12_** | -59.7386 | -82.2790 | -114.3485 | -122.9973 | -127.4181 |
| **Et_3_SiH** | -59.8615  (+.12) | -82.1638  (-.12) | -114.2627  (-.09) | -122.9455  (-.05) | -127.2988  (-.12) |
| ***i*-Pr_3_SiH** | -59.9143  (+.18) | -81.9074  (-.37) | -114.2034  (-.15) | -122.9049  (-.09) | -127.2062  (-.21) |

| **I–(CF_2_)_7_F** | | | | | | | |
| --- | --- | --- | --- | --- | --- | --- | --- |
|  | **I–C*F*_2_** | **I–(CF_2_)C*F*_2_** | **I–(CF_2_)_2_C*F*_2_** | **I–(CF_2_)_3_C*F*_2_** | **I–(CF_2_)_4_C*F*_2_** | **I–(CF_2_)_5_C*F*_2_** | **I–(CF_2_)_6_C*F*_3_** |
| ***c*-C_6_H_12_** | -59.6247 | -82.3331 | -114.1080 | -121.9849 | -123.0089 | -123.7992 | -127.3717 |
| **Et_3_SiH** | -59.7843  (+.16) | -82.2368  (-.10) | -114.0333  (-.07) | -121.9648  (-.02) | -122.9431  (-.07) | -123.7442  (-.06) | -127.3029  (-.07) |
| *i*-Pr_3_SiH | -59.7785  (+.15) | -81.9459  (-.39) | -113.9331  (-.17) | -121.8766  (-.11) | -122.8443  (-.16) | -123.6325  (-.17) | -127.1605  (-.21) |

| **(I–CF_2_)_2_** | |
| --- | --- |
|  | **(I–C*F*_2_)_2_** |
| ***c*-C_6_H_12_** | -53.1849 |
| **Et_3_SiH** | -53.2442  (+.06) |
| ***i*-Pr_3_SiH** | -53.2025  (+.02) |

| **(I–CF_2_CF_2_)_2_** | | |
| --- | --- | --- |
|  | **(I–C*F*_2_CF_2_)_2_** | **(I–CF_2_C*F*_2_)_2_** |
| ***c*-C_6_H_12_** | -59.3203 | -113.1787 |
| **Et_3_SiH** | -59.3925  (+.07) | -113.1229  (-.06) |
| ***i*-Pr_3_SiH** | -59.3956  (+.08) | -113.0819  (-.10) |

| **(I–CF_2_CF_2_CF_2_)_2_** | | | |
| --- | --- | --- | --- |
|  | **(I–C*F*_2_CF_2_CF_2_)_2_** | **(I–CF_2_C*F*_2_CF_2_)_2_** | **(I–CF_2_CF_2_C*F*_2_)_2_** |
| ***c*-C_6_H_12_** | -59.4508 | -114.0726 | -121.9749 |
| **Et_3_SiH** | -59.5342  (+.08) | -113.9619  (-.11) | -121.8741  (-.10) |
| ***i*-Pr_3_SiH** | -59.5241  (+.07) | -113.8915  (-.18) | -121.8323  (-.14) |

| **(I–CF_2_CF_2_CF_2_CF_2_)_2_** | | | | |
| --- | --- | --- | --- | --- |
|  | **(I–C*F*_2_CF_2_CF_2_CF_2_)_2_** | **(I–CF_2_C*F*_2_CF_2_CF_2_)_2_** | **(I–CF_2_CF_2_C*F*_2_CF_2_)_2_** | **(I–CF_2_CF_2_CF_2_C*F*_2_)_2_** |
| ***c*-C_6_H_12_** | -59.5322 | -114.1081 | -121.9359 | -122.7860 |
| **Et_3_SiH** | -59.6315  (+.10) | -113.9835  (-.12) | -121.8660  (-.07) | -122.6606  (-.13) |
| ***i*-Pr_3_SiH** | -59.6322  (+.10) | -113.9110  (-.20) | -121.8246  (-.11) | -122.6044  (-.18) |

**Table S3.** ^19^F NMR Chemical shifts of I–(CF_2_)_8_–I in *n*-hexane, *n*-pentane, cyclopentane, Et_3_SiH, and *i*-Pr_3_SiH. The shifts of ^19^F signals in trialkylsilanes with respect to the different saturated hydrocarbons used as reference solvents (Δδ=δ_reference hydrocarbon solvent_-δ_R3SiH_) are given in parenthesis.

| **(I–CF_2_CF_2_CF_2_CF_2_)_2_** | | | | |
| --- | --- | --- | --- | --- |
|  | **(I–C*F*_2_CF_2_CF_2_CF_2_)_2_** | **(I–CF_2_C*F*_2_CF_2_CF_2_)_2_** | **(I–CF_2_CF_2_C*F*_2_CF_2_)_2_** | **(I–CF_2_CF_2_CF_2_C*F*_2_)_2_** |
| ***n*-C_6_H_14_** | -59.3002 | -113.9955 | -121.9024 | -122.6865 |
| **Et_3_SiH** | -59.6315  (+.33) | -113.9835  (-.012) | -121.8660  (-.04) | -122.6606  (-.03) |
| ***i*-Pr_3_SiH** | -59.6322  (+.33) | -113.9110  (-.08) | -121.8246  (-.08) | -122.6044  (-.08) |
|  |  |  |  |  |
| ***n*-C_5_H_12_** | -59.3220 | -114.0486 | -121.9437 | -122.7324 |
| **Et_3_SiH** | -59.6315  (+.31) | -113.9835  (-.07) | -121.8660  (-.08) | -122.6606  (-.07) |
| ***i*-Pr_3_SiH** | -59.6322  (+.31) | -113.9110  (-.14) | -121.8246  (-.12) | -122.6044  (-.13) |
|  |  |  |  |  |
| ***c*-C_5_H_10_** | -59.6218 | -114.2284 | -122.1122 | -122.9281 |
| **Et_3_SiH** | -59.6315  (+.01) | -113.9835  (-.24) | -121.8660  (-.25) | -122.6606  (-.27) |
| ***i*-Pr_3_SiH** | -59.6322  (+.01) | -113.9110  (-32) | -121.8246  (-.29) | -122.6044  (-.33) |

**Table S4.** ^19^F NMR Chemical shifts of I–(CF_2_)_8_–I in cyclohexane and *i*-Pr_3_SiH/cyclohexane mixtures. The shifts of ^19^F signals in *i*-Pr_3_SiH/cyclohexane mixtures with respect to cyclohexane (Δδ=δ_cyclohexane_-δ*_i_*_-Pr3SiH/cyclohexane mixtures_) are given in parenthesis.

| **(I–CF_2_CF_2_CF_2_CF_2_)_2_** | | | | |
| --- | --- | --- | --- | --- |
|  | **(I–C*F*_2_CF_2_CF_2_CF_2_)_2_** | **(I –CF_2_C*F*_2_CF_2_CF_2_)_2_** | **(I –CF_2_CF_2_C*F*_2_CF_2_)_2_** | **(I –CF_2_CF_2_CF_2_C*F*_2_)_2_** |
| ***c*-C_6_H_12_** | -59.5322 | -114.1081 | -121.9359 | -122.7860 |
| ***i*-Pr_3_SiH : *c*-C_6_H_12_ (1:1)** | -59.5983  (+.07) | -113.9840  (-.12) | -121.8686  (-.07) | -122.6731  (-.11) |
| ***i*-Pr_3_SiH : *c*-C_6_H_12_ (1:2)** | -59.5744  (+.04) | -114.0035  (-.10) | -121.8713  (-.06) | -122.6906  (-.10) |

**4. Cartesian coordinates and Thermochemical data**

Thermochemical data at 298.15K and 0.1 MPa:

|  | Electronic Energy (H) | ZPE (kJ/mol) | chem.pot (kJ/mol) | internal thermal energy(kJ/mol) | entropy (kJ/molK-1) | enthalpy (kJ/mol) |
| --- | --- | --- | --- | --- | --- | --- |
| GeMe3H--BrCN | -4863.556538 | 327.8 | 220.62 | 357.78 | 0.46833 | 360.25 |
| GeMe3H--SeCN2 | -4783.7142 | 349.8 | 236.61 | 386.08 | 0.50964 | 388.56 |
| GeMe3H--AsCN3 | -4710.808821 | 371.3 | 241.02 | 419.03 | 0.60536 | 421.51 |
| GeMe3H---BF3 | -2521.283964 | 338.8 | 239.78 | 368.00 | 0.43835 | 370.48 |
| GeMe3H---COF2 | -2509.743112 | 344.3 | 227.01 | 379.83 | 0.52087 | 382.31 |
| GeMe3H---NO2F | -2501.615415 | 342.9 | 229.6 | 374.37 | 0.49386 | 376.85 |
| OC---BrCN | -2779.862355 | 36.85 | -43.59 | 54.77 | 0.3382 | 57.25 |
| OC---Se(CN)2 | -2700.018076 | 58.77 | -40.92 | 83.17 | 0.42452 | 85.65 |
| OC---As(CN)3 | -2627.109485 | 80.25 | -27.09 | 111.40 | 0.4728 | 113.88 |
| FH---BrCN | -2767.029265 | 53.62 | -20.61 | 67.78 | 0.30477 | 70.26 |
| FH---SeCN2 | -2687.182028 | 75.28 | -18.26 | 96.19 | 0.39219 | 98.67 |
| FH---AsCN3 | -2614.271438 | 96.35 | -6.85 | 124.32 | 0.44824 | 126.8 |

GeMe3H--AsCN3

Ge -2.9642206 1.0702622 0.0490749

H -1.7852444 1.7134613 -0.7678871

C -2.4663098 -0.7938114 0.3450481

H -2.3516800 -1.3144305 -0.6058094

H -1.5289104 -0.8620851 0.8971791

H -3.2443351 -1.2964850 0.9217000

C -3.0961534 2.0642837 1.7217309

H -2.1670299 1.9968588 2.2878422

H -3.3140308 3.1134259 1.5219360

H -3.9024004 1.6538256 2.3317423

C -4.5932864 1.2180190 -1.0080721

H -4.8482042 2.2639732 -1.1774339

H -4.4771831 0.7226960 -1.9715417

H -5.4167944 0.7459794 -0.4696789

C -2.0479738 2.2543296 -3.4640843

N -2.6061566 1.4775589 -4.1038763

C -0.7312027 4.7179208 -3.7194964

N -0.4202580 5.5112828 -4.4938982

As -0.9753889 3.3792944 -2.3554919

C -2.4402330 4.2914246 -1.5360059

N -3.2550005 4.8243655 -0.9223715

GeMe3H--BrCN

Ge -3.0536689 1.3407019 -0.0274461

H -2.5368508 2.0738335 -1.2959158

C -2.3821081 -0.4935841 -0.0641540

H -2.7371498 -1.0144043 -0.9539454

H -1.2917906 -0.5073865 -0.0678279

H -2.7304060 -1.0373691 0.8153977

C -2.3798250 2.2953225 1.5382080

H -1.2897208 2.3097121 1.5387751

H -2.7386892 3.3249584 1.5465211

H -2.7185652 1.8050621 2.4522157

C -5.0066139 1.3652713 -0.0684812

H -5.3767386 2.3909229 -0.0748485

H -5.3814355 0.8576422 -0.9577050

H -5.4072275 0.8597525 0.8115348

C -1.1165599 3.8177737 -5.1190046

N -0.7249346 4.2694348 -6.1045182

Br -1.7175949 3.1153577 -3.5897377

GeMe3H--SeCN2

Ge -2.8886783 1.4142311 -0.1820833

H -2.0748349 2.0613416 -1.3469638

C -2.3897009 -0.4729635 -0.1106999

H -2.6435006 -0.9731772 -1.0456960

H -1.3193415 -0.5854097 0.0639996

H -2.9249307 -0.9685232 0.7007145

C -2.3816410 2.3368123 1.4632312

H -1.3103132 2.2485014 1.6452807

H -2.6388135 3.3947673 1.4051293

H -2.9123001 1.9004563 2.3109992

C -4.7891003 1.6328598 -0.5520237

H -5.0475062 2.6884932 -0.6313954

H -5.0612670 1.1470882 -1.4887624

H -5.3740612 1.1891928 0.2553462

Se -1.4042485 3.2621406 -3.4741602

C -1.1126038 4.1834986 -5.0513995

N -0.8268799 4.7481235 -6.0149111

C -3.2375148 3.3490693 -3.5724608

N -4.3897788 3.3696994 -3.5757163

GeMe3H---BF3

B 0.0000000 0.0000000 -3.3557889

F 0.6566523 -1.1373551 -3.3941505

F 0.6566523 1.1373551 -3.3941505

F -1.3133045 0.0000000 -3.3941505

Ge 0.0000000 0.0000000 0.4832605

H 0.0000000 0.0000000 -1.0673904

C -1.8568560 0.0000000 1.0865049

H -1.8960691 0.0000000 2.1767858

H -2.3793325 0.8846034 0.7220831

H -2.3793325 -0.8846034 0.7220831

C 0.9284280 -1.6080845 1.0865049

H 0.4235772 -2.5028641 0.7220831

H 1.9557553 -1.6182607 0.7220831

H 0.9480346 -1.6420440 2.1767858

C 0.9284280 1.6080845 1.0865049

H 1.9557553 1.6182607 0.7220831

H 0.4235772 2.5028641 0.7220831

H 0.9480346 1.6420440 2.1767858

GeMe3H---COF2

C -0.8177020 0.7198460 -3.5825148

O 0.0185185 0.8400734 -2.7715579

F -1.7696654 1.5802444 -3.8362701

F -0.9496669 -0.2866330 -4.4067751

Ge -2.4307663 -0.5303774 -0.5169406

H -2.6720113 -0.5853637 -2.0501592

C -3.9726018 -1.3439170 0.3695643

H -3.8409877 -1.3127090 1.4522624

H -4.8888390 -0.8084279 0.1187367

H -4.0872580 -2.3851199 0.0665619

C -0.7993005 -1.5242313 -0.1092109

H -0.8876269 -2.5616875 -0.4331783

H 0.0537215 -1.0721942 -0.6155627

H -0.6112106 -1.5136681 0.9655355

C -2.2435979 1.3400468 0.0174795

H -1.3814112 1.7899656 -0.4748400

H -3.1347363 1.9098865 -0.2477121

H -2.1004146 1.4079734 1.0970462

GeMe3H---NO2F

N 3.2619620 -0.1053669 -5.0797863

O 4.4231027 -0.2423011 -5.1246533

O 2.5285929 0.8004047 -4.9784375

F 2.5611255 -1.3662252 -5.1752564

Ge 3.2870753 -0.3097446 -0.8121014

H 3.3142238 -0.3058568 -2.3587875

C 1.5777188 -1.0475258 -0.2162240

H 1.5352969 -1.0612181 0.8739614

H 0.7479206 -0.4442357 -0.5857035

H 1.4504895 -2.0679610 -0.5789325

C 4.7659836 -1.4151993 -0.1694759

H 4.6664966 -2.4388241 -0.5319917

H 5.7211450 -1.0185981 -0.5149092

H 4.7750862 -1.4351711 0.9213945

C 3.4869093 1.5296376 -0.1797072

H 4.4331753 1.9522119 -0.5189667

H 2.6758603 2.1550364 -0.5543102

H 3.4681191 1.5578612 0.9109758

OC---BrCN

C -1.0693328 3.8741592 -5.2348541

N -0.7189769 4.3378679 -6.2302435

Br -1.6128212 3.1569218 -3.6935023

C -2.6263080 1.8870102 -0.9083547

O -2.9924344 1.4592414 0.0626795

OC---SeCN2

Se -1.2969216 3.4416123 -3.7264262

C -1.0865542 4.3377977 -5.3232296

N -0.8551379 4.8892457 -6.3085280

C -3.1345678 3.4640617 -3.7508726

N -4.2852642 3.4360363 -3.6933778

C -2.1771677 1.9051382 -1.0621102

O -2.5404795 1.3582487 -0.1524723

OC---AsCN3

C -1.9507691 2.3998537 -3.5787580

N -2.5028533 1.5791314 -4.1670271

C -0.7115711 4.9008210 -3.9228327

N -0.4588133 5.6893436 -4.7224157

As -0.8757483 3.5867470 -2.5404967

C -2.3384034 4.4532992 -1.6733795

N -3.1428824 4.9647783 -1.0286566

C -1.9975170 1.4336680 -0.4712310

O -2.4356420 0.6749661 0.2284531

FH---BrCN

C -1.0766476 3.8655803 -5.2334719

N -0.6954972 4.3154281 -6.2198591

Br -1.6665849 3.1728117 -3.7098273

F 0.1591886 5.4816286 -8.5306381

H -0.1302514 5.0735321 -7.7400311

FH---SeCN2

Se -1.3940328 3.2910699 -3.5352584

C -1.1144084 4.2184786 -5.0962098

N -0.8519551 4.7901158 -6.0579534

C -3.2282491 3.3640815 -3.6006709

N -4.3796457 3.3581794 -3.5556701

H -0.4771393 5.7243808 -7.5623300

F -0.2794135 6.2113550 -8.3340854

FH---AsCN3

H 0.0007469 6.8306673 -5.7408207

C -2.0368330 2.2764193 -3.4828513

N -2.5981034 1.4770545 -4.0915404

C -0.7248192 4.7479019 -3.7469674

N -0.4450273 5.5456419 -4.5229195

As -0.9646557 3.4132374 -2.3937172

C -2.4269005 4.3040275 -1.5590909

N -3.2397080 4.8188751 -0.9276147

F 0.2562541 7.4870859 -6.3519515

**5. References**

22. Ahlrichs, R., Bär, M., Häser, M., Horn, H. & Kölmel, C. Electronic structure calculations on workstation computers: The program system turbomole. *Chem. Phys. Lett.* **162**, 165–169 (1989).

23. Adamo, C. & Barone, V. Toward reliable density functional methods without adjustable parameters: The PBE0 model. *J. Chem. Phys.* **110**, 6158–6170 (1999).

24. Caldeweyher, E., Ehlert, S., Hansen, A., Neugebauer, H., Spicher, S., Bannwarth, C. & Grimme, S. A generally applicable atomic-charge dependent London dispersion correction. *J. Chem. Phys.* **150**, 154122 (2019).

25. Dunning, T. H. Jr. Gaussian basis sets for use in correlated molecular calculations. I. The atoms boron through neon and hydrogen. *J Chem. Phys.* **90**, 1007–1023 (1989).

26. B. Mallada; A. Gallardo; M. Lamanec; B. De La Torre; V. Špirko; P. Hobza; P. Jelinek. Real-space imaging of anisotropic charge of σ-hole by means of Kelvin probe force microscopy. *Science* **2021**, *374*, 863–867.

27. Becke, A. D. & Edgecombe, K. E. A simple measure of electron localization in atomic and molecular systems. *J. Chem. Phys.* **92**, 5397–5403 (1990).

28. Bader, R. F. W. A quantum theory of molecular structure and its applications. Chem. Rev. **91**, 893–928 (1991).

29. Lu, T. & Chen, F. Multiwfn: A multifunctional wavefunction analyzer. *J. Computational Chem.* **33**, 580–592 (2012).

30. Bartashevich, E., Mukhitdinova, S., Yushina, I., Tsirelson, V. Electronic Criterion for Categorizing the Chalcogen and Halogen Bonds: Sulfur–Iodine Interactions in Crystals. *Acta Crystallogr. Sect. B Struct. Sci. Cryst. Eng. Mater.* **75**, 117–126 (2019).

31. Glendening, E. D., Landis, C. R. & Weinhold, F. Natural bond orbital methods. *WIREs Comput. Mol. Sci.* **2**, 1–42 (2012).

32. E. D. Glendening, J. K. Badenhoop, A. E. Reed, J. E. Carpenter, J. A. Bohmann, C. M. Morales, P. Karafiloglou, C. R. Landis and F. Weinhold, NBO 7.0, 2018.

33. Humphrey, W., Dalke, A. & Schulten, K. VMD: Visual molecular dynamics, *J. Molecular Graph.* **14**, 33–38 (1996).

34. Metrangolo, P.. Panzeri, W., Recupero, F., Resnati, G. ^19^F NMR Study of the Halogen Bonding between Haloperfluorocarbons and Heteroatom containing Hydrocarbons *J. Fluorine Chem.* **114**, 27–33 (2002).
